# Understanding Immune Evasion and Therapeutic Targeting Associated with PD-1/PD-L1 Pathway in Diffuse Large B-cell Lymphoma

**DOI:** 10.3390/ijms20061326

**Published:** 2019-03-15

**Authors:** Moo-Kon Song, Byeong-Bae Park, Jieun Uhm

**Affiliations:** 1 Department of Hematology-Oncology, Hanyang University Hanmaeum Changwon Hospital, Changwon 51497, Korea; song9676@hanmail.net; 2 Division of Hematology-Oncology, Department of Internal Medicine, Hanyang University College of Medicine, Hanyang University Seoul Hospital, Seoul 04763, Korea; jieunuhm@hanyang.ac.kr

**Keywords:** diffuse large B cell lymphoma, PD-1, immune checkpoint

## Abstract

In tumor microenvironment, the programmed death 1 (PD-1) immune checkpoint has a crucial role of mechanism of T cell exhaustion leading to tumor evasion. Ligands of PD-1, programmed death ligand 1/2 (PD-L1/L2) are over-expressed in tumor cells and participate in prolonged tumor progression and survivals. Recently, clinical trials for patients who failed to obtain an optimal response prior to standardized chemotherapy in several solid cancers have been focused on targeting therapy against PD-1 to reduce disease progression rates and prolonged survivals. Since various inhibitors targeting the immune checkpoint in PD-1/PD-L1 pathway in solid cancers have been introduced, promising approach using anti-PD-1 antibodies were attempted in several types of hematologic malignances. In diffuse large B cell lymphoma (DLBCL) as the most common and aggressive B cell type of non-Hodgkin’s lymphoma, anti-PD-1 and anti-PD-L1 antibodies were studies in various clinical trials. In this review, we summarized the results of several studies associated with PD-1/PD-L1 pathway as an immune evasion mechanism and described clinical trials about targeting therapy against PD-1/PD-L1 pathway in DLBCL.

## 1. Introduction

Diffuse large B-cell lymphoma (DLBCL) is the most common and aggressive type of B-cell lymphoma, accounting for 30–35% of non-Hodgkin’s lymphoma (NHL) [1]. It can be divided into germinal center (GC) type and non-GC type according to a cell or origin, which is identified by gene expression profile or Hans algorithm [2]. Thus, DLBCL has heterogeneous genetic features and clinical outcomes similar to those of other NHLs. Although about 60% of the patients with DLBCL can be cured after introduction of CD 20 monoclonal antibody (mAb) rituximab, 30–40% of those will experience a relapse or be refractory to standard therapy [3], which suggests the need for more potent and effective therapies in high-risk patients.

Recently, our understanding of immune evasion mechanisms in tumor progression and dissemination over time was developed. Further investigations led to recognition of the clinical importance of checkpoints in the immune evasion mechanism associated with T-cell exhaustion and protection of target cells. In detail, cytotoxic T-lymphocyte-associated antigen 4 (CTLA-4)/B7-1 or B7-2 and programed cell death 1 (PD-1)/its ligands programed cell death 1 ligand 1 (PD-L1) or PD-L2 axis are recognized to be the most important central checkpoints in the immune invasion for tumor progression [4].

CTLA-4 is a checkpoint inhibitor, which stops naive T-cell activation in the initial stage and thus inhibits the anti-tumor immune response [5]. CTLA-4 blockade using mAb ipilimumab improves the anti-tumor response. However, there are not enough clinical data on ipilimumab to explain the anti-tumor efficacy in DLBCL.

PD-1 expressed on T cells binds to PD-L1/L2 on tumor cells or other target cells, and then a downstream signaling pathway leads to T-cell exhaustion and protection of tumor cells [6]. The field of clinical trials in NHL including DLBCL associated with PD-1/PD-L1 blockade is being actively studied and is drawing promising results. This review focuses on understanding the immune evasion mechanism associated with the PD-1/PD-L1 signaling pathway as well as clinical application of mAb against PD-1 and its ligands in DLBCL.

## 2. Structural Characteristics of PD-1 and the Ligands, PD-L1/L2 

PD-1 is expressed on T-cells as a monomer and is a negative regulator of interleukin-2 (IL-2) production and T-cell proliferation [7,8]. Structurally, PD-1 is a protein encoded by the 5-exon PDCD1 gene chromosome 2q37.3. It contains a single immunoglobulin (Ig) V-like domain, a transmembrane domain, and an intracellular domain with an immunoreceptor tyrosine-based inhibitory motif (ITIM) and an immunoreceptor tyrosine-based switch motif (ITSM) [9,10]. 

As a PD-1 ligand, PD-L1/L2 contains an extracellular IgV and IgC domain and a transmembrane domain, but it lacks an identifiable intracellular-signaling domain [11]. PD-L1 and PD-L2 share 37% identify but differ in their affinity for PD-1 and expression patterns [12]. Moreover, PD-L1 is widely expressed in both hematopoietic and non-hematopoietic cells, whereas PD-L2 is expressed in activated dendritic cells and some macrophages. Especially, PD-L1 is encoded by the 8-exon CD274 gene on chromosome 9p24.1. Unlike PD-L1, expression of PD-L2 has not been documented, because it is not expressed in most NHL cell lines [13]. Moreover, a recent study demonstrated that low PD-L2 expression in DLBCL cell lines and the difference between subtypes is not present [14]. Thus, description of PD-L2 expression is excluded in this review.

## 3. T-Cell Exhaustion by the PD-1 Signaling Pathway

Naïve T cells are activated after recognizing an antigen through interaction between T-cell receptors (TCR) and major histocompatibility complex molecules on antigen-presenting cells. The activating signals are modulated by checkpoint molecules as inhibitory receptors. The checkpoint molecule has a crucial role in inhibiting the T-cell-mediated immune response and maintaining self-tolerance in chronic infectious or inflammatory conditions [15]. Similarly, evading immune surveillance by inhibitory signals through active interactions between checkpoint molecules and ligands eventually promotes T-cell exhaustion and tolerance in cancerous status. 

**Figure 1:**
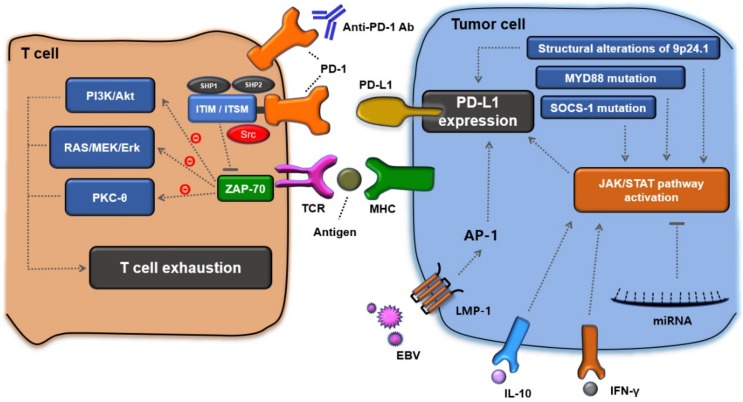
Immune evasion mechanisms associated with the PD-1/PD-L1 signaling pathway in the tumor microenvironment of lymphoma. Upon PD-1 engagement, SHP-1/2 is recruited and then the downstream signal of TCR is inhibited. Ultimately, T-cell exhaustion and tolerance is induced. Meanwhile, PD-L1 expression is promoted via multiple mechanisms, such as alterations of chromosome 9p24.1, MYD88 mutation, SOCS-1 mutation, EBV infection, and increased cytokines (IFN-γ, IL-10); hence cancer-cell proliferation and dissemination is possible.

In a tumor microenvironment, PD-1 expressed on the surface of activated T-cells by downstream signaling of TCR, has an important checkpoint function in regulating T-cell-mediated anti-tumor immune responses. It delivers inhibitory signals to regulate T-cell activation after binding with the ligand, PD-L1 (Figure 1). Interaction between extracellular domains of PD-1 and PD-L1 induces a conformational change of PD-1 resulting in phosphorylation of cytoplasmic ITIM and ITSM, which subsequently recruit Src Homology Region 2-Containing Protein *Tyrosine* Phosphatase-2 (SHP-2) and SHP-1 [16,17]. Once SHP-1/2 is recruited, it dephosphorylates ζ-associated protein 70 (ZAP70) as a downstream member of TCR signaling pathways and thus inhibits the phosphatidylinositol-3-kinase/Akt (PI3K/Akt) pathway, RAS/MEK/Erk pathway, and protein kinase C-θ (PKC-θ) [17,18]. Ultimately, the PD-1-mediated inhibitory pathway is closely associated with decreasing T-cell proliferation and IL-2 production, and promoting T-cell apoptosis, leading to T-cell exhaustion. 

## 4. Immune Evasion Mechanisms for PD-L1 Expression in Lymphoma Cells 

Structural alterations such as amplifications, gains, and translocations of chromosome 9p24.1 directly increase expression of PD-L1 [19,20]. Moreover, the alterations of 9p24.1 induce Janus Kinase 2 (JAK2) amplification leading to augmentation of JAK/Signal Transducers and Activators of Transcription (STAT) signaling, which induces PD-L1 expression as an extra-signaling pathway [20]. Increased IL-10 can induce tyrosine phosphorylation of JAK2 and STAT3 [21,22]. Then, the activated JAK/STAT pathway eventually induces over-expression of PD-L1 (Figure 1).

PD-L1 is also regulated by the interferon gamma (IFN-γ) receptor singling pathway. In the tumor microenvironment, IFN-γ produced by tumor-infiltrating lymphocytes (TILs) augments the JAK/STAT pathway by activating the receptors [23,24]. PD-L1 expression is eventually upregulated by the activated JAK/STAT pathways.

Suppressor of cytokine signaling 1 (SOCS1) is a postulated tumor suppressor gene associated with growth arrest of tumor cells, rapid dephosphorylation of JAK2, and silencing of cyclin D1 [25,26]. However, mutations of the C-terminal domain including SOCS box, which is necessary for the inhibitory function, result in activation of the downstream JAK/STAT pathway and subsequent upregulation of PD-L1 expression [27,28]. 

MicroRNAs (miRNAs) have a crucial role in regulating the expression of oncogenes and function as tumor suppressors to target JAK2 [29,30,31]. Thus, increased levels of miRNAs induce downregulation of the JAK2 protein, thus promoting apoptosis and inhibiting proliferation of tumor cells by downregulating the anti-apoptotic protein, Bcl-xL. Moreover, miRNAs are thought to directly bind with the 3′-untranslated region (3′UTR), which is a crucial determinant of PD-L1 expression and then inhibits the expression [32,33,34]. For instance [35], miR-142-5p could inhibit growth of pancreatic cancer cells; miR-187 inhibits osteosarcoma cells; miR-424 could regulate the chemoresistance of epithelial ovarian cancer via T cells; miR-135a is associated with regulation of classic Hodgkin’s lymphoma cells; miR-195 is tumor suppressor gene which is associated with cell growth in several cancers. Decreased levels of miRNAs might be a clinical predictor of disease progression or relapse in cancer.

An intrinsic signal by Epstein–Barr virus (EBV) infection augments PD-L1 expression on tumor cells and infiltrating macrophages [20,36]. EBV latent membrane protein 1 (LMP-1) induces activation of the transcription factor, activator protein 1 (AP-1), by activating the c-Jun N-terminal kinase (JNK) cascade [37,38]. In this manner, the JAK/STAT pathway is activated and then PD-L1 expression is augmented. 

Myeloid differentiation primary response gene 88 (MYD88) is an adaptor protein that participates in the innate immune response and plays an important role in the homeostasis of human B cells [39]. However, once MYD88 mutates, it phosphorylates IL-1 receptor-associated kinase after toll-like receptor activation and subsequently activates nuclear factor kB [40,41]. Then, it activates the JAK/STAT signaling pathways and ultimately upregulates PD-L1 expression in lymphoma cell lines [42]. 

## 5. Immune Evasion Mechanisms to Augment PD-L1 Expression in DLBCL

Genetic anomalies or chromosomal alterations leading to PD-L1 expression were observed in about 20% of DLBCL [43,44]. Particularly, structural alterations of 9p24.1 were closely associated with PD-L1 expression in DLBCL. Recently, Georgiou et al. reported that the genetic alterations such as 12% of gains, 3% of amplifications, and 4% of translocations were observed and other translocations involving Ig heavy chain locus could also lead to PD-L1 expression in DLBCL [19]. The data showed that these cytogenetic alterations correlated with increased PD-L1 expression. Moreover, they found that genetic alterations in the PD-L1 locus are mainly present in non-GC type of DLBCL, but not GC type. The data suggested that treatments targeting PD-1/PD-L1 signaling pathway might be more benefit in patients with non-GC type than those with GC type.

EBV also provides clinical evidence that the intrinsic signal by viral infection augments PD-L1 expression in DLBCL [21,45]. As mentioned above, induction of PD-L1 expression as an immune escape signal by constitutive signaling through EBV-encoded protein LMP1 and AP-1 could be activated by JNK cascade and contribute lymphomagenesis in EBV-positive DLBCL. 

Deregulated inflammatory cytokines could contribute to DLBCL cell growth. Recently, an aberrant JAK/STAT activating pathway as a downstream of the IL-10 signaling was found in DLBCL cell lines and it is known to directly augment PD-L1 overexpression [22,46].

Another intrinsic mechanism associated with PD-L1 expression in DLBCL cells is gene regulation by miR-195, which is thought to bind with the 3′UTR of the PD-L1 protein and then inhibit the expression. In recent data, miR-195 was significantly decreased, whereas PD-L1 as the downstream gene was highly expressed in DLBCL cell lines [35].

PD-L1 expression in non-GC-type DLBCL can also result from activation of JAK/STAT signaling by MYD88 mutations [41,47,48]. The mutations have been found in up to 30% of the non-GC type in DLBCL cases, and the MYD88 L265P mutation is found to be the most common oncogenic type.

## 6. Prognostic Values of PD-1, PD-L1, and Soluble PD-L1 Expression in DLBCL

Several studies have demonstrated an association between the PD-1/PD-L1 axis and prognosis in DLBCL. The data which quantified the prevalence of PD-1^+^ expression in DLBCL showed that it has ranged from 39.5% to 68.6% [43,49,50,51,52,53]. Although there were some unmatched findings because of a variety of immunohistochemistry stains and cut-off values of PD-1/PD-L1 positivity, numerous studies have showed that a high number of PD-1^+^ TILs in DLBCL is associated with favorable clinical features and prognosis (Table 1) [43,49,50,51].

Interestingly, these results are in contrast to data in solid tumors that the number of PD-1^+^ TILs positively correlates with tumor-specific PD-L1 expression and is a poor prognostic factor [62,63]. Moreover, Kiyasu et al. observed that a low number of PD-1^+^ TILs was associated with PD-L1^+^ and microenvironmental PD-L1^+^ (defined as PD-L1 in non-malignant cells; mPD-L1) in 236 patients with DLBCL [43]. They also found that patients with a low number of PD-1^+^ TILs and PD-L1^+^ DLBCL have worse outcomes than do those with PD-L1^−^ or mPD-L1^−^. Because PD-1^+^ is expressed at high levels on GC follicular helper T cells in normal tissue, PD-1^+^ TILs are abundant in GC type DLBCL. Thus, the number of PD-1^+^ TILs might be associated with not only tumor-mediated T cell exhaustion but also lymphoma cell origin. 

Similarly, the presence of a high number of PD-1^+^ TILs in follicular lymphoma was a favorable prognostic factor, whereas a low number of TILs was associated with increased risk of histologic transformation [64,65]. Although engaged PD-1 on T cells is known to suppress anti-tumor response, increased PD-1 on already activated T cells paradoxically reflects an active immune response to tumor cells. Thus, PD-1 may be considered to be a favorable prognostic factor in DLBCL. However, several data of PD-1 in Table 1 were confused due to inconsistent staining technique and diverse the cut-off value. Further, well-designed investigation will be needed to confirm the clinical significance of PD-1.

PD-1 can inhibit T cell activation after interaction between PD-1 and its ligands and thus clinical value of PD-1 ligand, PD-L1 in tumor cells is also important. PD-L1 was also expressed in a variety of prevalence and more frequently in non-GC-type DLBCL. Andorsky et al. found that the PD-L1^+^ DLBCL cell lines have characteristics consistent with the non-GC type [13]. This finding suggests that PD-L1 expression in DLBCL might reflect aggressive clinical features. Moreover, Kiyasu et al. analyzed 1253 DLBCL samples using PD-L1 and PAX5 double staining technique [43]. In the data, 10.5% of the patients was PD-L1^+^ and 15.3% of those was mPD-L1^+^. Notably, group with PD-L1^+^ was significantly associated with poorer overall survival than those with PD-L1^−^. Moreover, PD-L1^+^ was closely associated with B symptoms, high International Prognostic Index risk group and non-GC type. Meanwhile, mPD-L1 positivity did not correlate with the survivals [43]. Most studies also showed comparable data that PD-L1 expression is associated with the aggressive and non-GC type and poor prognosis (Table 1) [43,54,55,56,57]. However, a data by Fang et al. was showed different result that PD-L1 expression is associated with prognostic significance in univariate analysis, but not multivariate analysis [51]. 

EBV provides intrinsic signal by LMP1 to augment PD-L1 expression in DLBCL. Therefore, EBV^+^ DLBCL cells could have high PD-L1 and mPD-L1 expressions and thus it is assumed that EBV^+^ DLBCL patients has a poorer prognosis than EBV^−^ those. However, clinical significance of the expressions of PD-L1 and mPD-L1 is currently unclear [66]. 

PD-1 plays a certain role in peripheral tolerance and homeostasis by inhibiting T-cell activation through interaction with PD-L1 expressed on tumor cells and non-malignant microenvironmental cells (MECs) activating the checkpoint pathway associated with tumor evasion mechanism [67]. Thus, recent studies investigated soluble PD-L1 (sPD-L1) in plasma, collected at the time of diagnosis of the disease, to evaluate the immune checkpoint effect on survival in patients with DLBCL (Table 1) [58,59,60,61]. Mostly, elevated levels of sPD-L1 in plasma correlated with decreased CR rates and poorer survivals in DLBCL patients even with high-dose chemotherapy [58,59,60], despite the negative result of data by Keane et al. [61]. Interestingly, Rossille et al. found no correlation between PD-L1 expression and sPD-L1 levels in patients with DLBCL [58], which suggests that the sPD-L1 level correlates with the degree of host immune response rather than with anti-tumor activity. Most clinical data of sPD-L1 demonstrated that elevated sPD-L1 is significantly associated with worse prognosis in DLBCL. However, there are only a few studies about sPD-L1 and comparative study by Keane et al. with other factors did not showed the independent prognostic significance [61].

## 7. Clinical Data of PD-1 and PD-L1 Blockade in DLBCL

Because about 30–40% of patients with DLBCL already have a relapse or refractory disease after standard therapy, novel therapeutic strategy is needed to increase response rate and prolong survivals. As mentioned above, PD-1/PD-L1 signaling pathway is closely associated with downregulation of cytotoxic T cell function and prolongation of DLBCL survivals. Therefore, PD-1/PD-L1 immune blockade by mAb could repair the exhausted T cell functions and augment anti-tumor activities. Recently, immunotherapy using anti-PD-1 and PD-L1 mAbs is considered to be a reasonable treatment in the patients with relapsed or refractory DLBCL. Actually, it has been validated that immunotherapy has a durable response and improved survival rates in several solid cancers and hematologic malignancies. Recently, clinical trials using several developed anti-PD-1 and anti-PD-L1 mAbs are actively conducted in DLBCL.

Pidilizumab was thought to be a first humanized IgG1 blocking mAb against PD-1. Therefore, several clinical trials to evaluate efficacy of the humanized anti-PD-1 mAb in DLBCL were performed. However, recent evidence suggests that pidilizumab is not an anti-PD-1 mAb, but an mAb against Delta-like ligand [66,68]. 

Nivolumab is a fully humanized IgG-4 blocking monoclonal antibody that targets the PD-1 receptor on human T-cells [69]. The anti-PD-1 mAb binds specifically to PD-1, but not to related members of CD28 family such as CD28, CTLA-4, inducible co-stimulator, and B or T lymphocyte attenuator. The blockade of PD-1 signaling pathway using nivolumab led to a reproducible enhancement of both proliferation of lymphocytes and release of IFN-γ [70]. The anti-PD-1 mAb had effective and safety profiles in the patients with in studies that involved patients with several types of cancer. Treatment-related adverse effects (AEs) were in most low grades and the incidence of drug associated severe or life-threatening AEs was low among several disease cohorts [71,72,73]. 

In a phase-1b dose-escalation cohort expansion study of nivolumab by Lesokhin et al., 81 patients with relapsed or refractory hematologic malignancies including 54 patients with NHLs (11 DLBCL patients) and 27 those with multiple myeloma were treated with two different dose levels of nivolumab (1mg/kg and 3 mg/kg every two weeks) (Table 2) [74]. Although overall response rate (ORR) among other B-cell NHL was 28%, ORR was higher in the patients with relapsed or refractory DLBCL (36%, 4 of 11 patients). From the DLBCL patients achieved the response, two achieved complete response (CR) and two more had a partial response (PR). After median follow-up of about six months, only one patient had a sustained response (77.3 weeks). Even though nivolumab-related AEs were present in 96%, AEs above than grade 3 was only 22%, and treatment was discontinued for 12% because of AEs. Nivolumab was well tolerated and more effective in relapsed or refractory DLBCL than were other B-cell NHLs.

A recently single-arm open-labeled phase II study for efficacy and safety of nivolumab in 121 patients with relapsed or refractory DLBCL who were ineligible for autologous stem cell transplantation (ASCT) and those experienced failure with ASCT was performed (Table 2) [75]. Nivolumab treatment was 3mg/kg every two weeks. Among the 121 enrolled patients, 87 patients in the ASCT-failed cohort received median of 4 doses of nivolumab, while 34 those in the ASCT-ineligible cohort received 3 doses. At a median follow-up of 9 months in ASCT-failed cohort, ORR were 10%, whereas ASCT-ineligible cohort achieved 3% ORR during median six-month follow-up. Median response duration was 11 months in ASCT-failed cohort and 8 months in ASCT-ineligible cohort. The median progression-free survival (PFS) was 1.9 months and median overall survival (OS) was 12.2 months in the ASCT-failed cohort, but median PFS was 1.4 months and median OS was 5.8 months in the ASCT-ineligible cohort. In the data, treatment related grade 3 or 4 AEs were present in 24% and the most common AEs were neutropenia (4%), thrombocytopenia (3%) and elevated pancreatic enzyme, lipase (3%). Collectively, nivolumab monotherapy had well tolerated AEs but a low ORR in both the ASCT-failed and the ineligible DLBCL cohorts. 

Pembrolizumab is also a humanized IgG-4 anti-PD-1 mAb with high affinity [69]. The target is PD-1 receptor on human T cells. It was demonstrated to have a robust antitumor activity and a favorable safety profile in various cancer types and is approved worldwide for advanced malignant neoplasms [77,78].

In a recent phase-2 study designed to evaluate the efficacy and safety of pembrolizumab as a humanized PD-1 mAb, 26 patients with relapsed chronic lymphocyte leukemia (CLL) (*n* = 16) and Richter’s transformation (RT) (*n* = 9, all were proven DLBCL) as transformed CLL were enrolled [76] (Table 2). The patients received pembrolizumab, a dose of 200 mg every three weeks. 60% of the patients were treated with ibrutinib. In 4 of 9 patients with RT, objective responses were present (ORR, 44%). One of 9 patients (11%) were achieved CR and 2 of 9 those (22%) were achieved PR. Meanwhile, patients with relapsed CLL did not achieved the response (ORR, 0%). Interestingly, all responses were observed in patients with RT who experienced disease progression after ibrutinib therapy. During a median follow-up time of 11 months, median PFS was 5.4 months and the OS was 10.7 months in RT patients. Treatment-related grade 3 or 4 AEs were found in 60% (*n* = 15) and were manageable. In this study, pembrolizumab exhibited to have a more selective treatment efficacy in CLL patients with RT than those with relapsed CLL.

Durvalumab is a selective, high-affinity, human IgG1 mAb against PD-L1 [79]. It binds to PD-L1 receptor on tumor cells and thus inhibit interaction between PD-1 and PD-L1 leading to recovery of cytotoxic function of T-cells. The United States of America Food and Drug Administration first approved breakthrough therapy designation to the anti-PD-L1 mAb in February 2016 for patients with inoperable or metastatic PDL1-positive urothelial bladder cancer [80]. In the field of immunotherapy in DLBCL, recent various phase-I/II studies in DLBCL were ongoing to investigate the efficacies of durvalumab monotherapy and durvalumab therapy combined with other agents (NCT02549651, NCT03212807, NCT03241017, NCT03003520, NCT02401048, and NCT2706405). 

Atezolizumab is also a humanized IgG1 mAb against PD-L1 [81]. It binds to PD-L1 and prevents the interaction between PD-L1 and its receptors PD-1 and CD80. The Fc region of atezolizumab is engineered to reduce function of Fc effector and minimize antibody-dependent cell-mediated cytotoxicity [76]. This prevents antibody-mediated hypothetical loss of PD-L1 expressing T cells and thus antitumor activity is enhanced. Therefore, it interrupts PD-1/PD-L1 axis, and thus prevents T cell exhaustion, downstream inhibition of cytokines, and late phase immune response. 

In addition, avelumab as a humanized IgG1 mAb against PD-L1 was showed that it leads to potent cell killing in the presence of natural killer cells purified from either healthy donors or cancer patients [82]. Like to durvalumab, early phase studies including atezolizumab (NCT2926833, NCT03422523, NCT02596971, NCT03321643, NCT0279896, NCT02220842, and NCT03276468) or avelumab (NCT03244176, NCT02951156, and NCT03440567) was also ongoing to evaluate efficacy and safety of the treatments in high-risk patients with DLBCL. 

However, it was recently reported that hyperprogressive disease was developed in 9% of patients received anti-PD-1/PD-L1 therapy [83,84]. The hyperprogression was associated with elderly age of patients but not tumor burden or cancer type [83]. MDM2/MDM4 amplification and EGFR aberration correlated with higher risk of hyperprogression in solid cancers [84], although it was little known in lymphoma. A recent data showed that patients experienced hyperprogression have a higher prevalence of PD-L1^−^ disease than those did not experienced the disease [83]. It is predicted to be because engagement of PD-1 with anti-PD-1 mAb inhibits but not augment T cell activations. Therefore, anti-PD-1 mAbs might be PD-1 agonist than antagonist in PD-L1^−^ status. Disease might also be rapidly progressed through interaction between PD-L1 and CD80 instead of PD-1 for the blocking duration by anti-PD-1 mAbs in PD-L1^+^ status [85]. Some polymorphism of PD-1 could also affect the action of anti-PD-1 mAbs and thus hyperprogression could be possible after PD-1 blockade [86].

## 8. Lack of Biomarker of Immunotherapy in DLBCL 

Biomarkers have various clinical applications including risk stratification, disease screening, determination of prognosis, prediction of response to chemotherapy, and monitoring of progression in cancers. Therefore, the establishment of useful biomarkers is of great importance to improve efficacy of chemotherapy. However, robust biomarker to assess response and predict prognosis is still absent in DLBCL patients received immunotherapy. As mentioned above, although PD-L1 expression was closely associated with prognosis in patients with DLBCL received chemotherapy in several clinical data, correlation between PD-1 blockade and PD-L1 expression in DLBCL is still inconclusive and controversial [61]. The discrepancy is because different detecting antibodies, staging techniques, and variable cut-off values of PD-L1 expression. 

Microsatellite instability (MSI) was emerged as an alternative biomarker to predict the response of PD-1 blockade in cancer. In clinical studies of PD-1/PD-L1 blockade in solid cancer, high MSI phenotype showed durable responses to PD-1 blockade [87,88]. The results led to clinical possibility of MSI as biomarker predictive for response to immunotherapy regardless of other characteristics of cancer. However, MSI status failed to show correlation with clinical response in CLL with RT. 

Structural alterations of PD-L1 as another possible biomarker were also studies. However, it did not show to be associated with PD-L1 expression or ORR in nivolumab study [74]. 

## 9. Conclusions 

In this article, we reviewed and discussed immune invasion mechanisms and clinical data of target therapies associated with PD-1/PD-L1 signaling pathways in DLBCL. In the tumor microenvironment, PD-1 expressed on T cells might play a crucial role as an important checkpoint to induce T-cell exhaustion and tolerance in DLBCL. Moreover, PD-L1 expressed on tumor cells is importantly associated with survivals and proliferations of the malignant B cells. 

However, it is somewhat disappointing that several clinical data about PD-1 expression in DLBCL did not demonstrate consistent the prognostic significance. Meanwhile, PD-L1 expression in DLBCL cell lines was shown to be closely associated with poor prognosis in DLBCL in most clinical data [43,54,55,56,57]. Entirely, it is speculated that PD-L1 expression in DLBCL has a better definite meaning than PD-1 expression. However, to analyze more precise clinical significances of PD-1 and PD-L1 expressions in DLBCL, the urgent problem to resolve is that each study should adopt consistent research method including unified staining method and cut-off value about PD-1 and PD-L1 expressions.

Recently, improved understanding of the PD-1/PD-L1 signaling pathway is leading to development of immunotherapy in patients with NHL including DLBCL. Phase I and II trials using anti-PD-1 mAbs such as nivolumab and pembrolizumab have been showed promising results and acceptable adverse effects in patients with relapsed or refractory DLBCL. Recently, several early phase clinical trials using anti-PD-L1 mAb such as durvalumab, atezolizumab, and avelumab to kill the resistant malignant lymphoma cells is ongoing. If the data from recent ongoing clinical studies of the immunotherapies are accumulated, better therapeutic targeting associated with PD-1/PD-L1 pathways will be available for patients with DLBCL. Further development of an immunotherapeutic strategy is expected to provide more improved clinical outcomes.

## Figures and Tables

**Table 1:** Summarized data associated with PD-1/PD-L1/sPD-L1 expression in DLBCL

Study (References)	No.	Methodology (Used Antibody)	Cut-Off Value	Frequency	Clinical & Prognostic Comments
**Data associated with PD-1 expression**					
Ahearne M.J. [49]	70	ND	≥ median value	PD-1^+^ cells, 50%	PD-1^+^ cells were associated with better OS. The cell numbers positively correlated with numbers of CD4^+^ cells.
Kiyasu J. [43]	236	NAT105 (Abcam)	≥ median value	PD-1^+^ cells, 50%	PD-1^+^ TILs were lower in patients with B symptoms, EN sites, bulky mass, non-GC type and PD-L1^+^ DLBCL.
Kwon D. [50]	126	MRQ-22 (Cell Marque)	≥ 1 PD-1^+^ cell	PD-1^+^ cells, 68.6%	PD-1^+^ TILs were associated with better PFS and OS. Number of the TILs positively correlated with PD-L1^+^ and mPD-L1^+^.
Fang X. [51]	76	MRQ-22 (ZSGB-BIO)	PD-1 expression	PD-1^+^ cells, 39.5%	PD-1^+^ TILs were associated with better OS.
Muenst S. [52]	184	Polyclonal Ab. (AF1086)	> 168/mm^2^ or 2.8%	ND	PD-1^+^ TILs were not significantly associated with survivals.
Cohen M. [53]	102	(AbD Serotec)	PD-1^+^ cell ≥ 13.1%	PD-1^+^ cells, 50%	High PD-1 cell numbers were associated with poor EFS.
**Data associated with PD-L1 expression**					
Siddiqi N. [54]	52	(Abcam EPR1161, 28-8 & SP263)	ND	ND	Higher PD-L1 expression in tumor cells correlated with more aggressive disease
Xing W. [55]	86	(E1L3N)	PD-L1^+^ ≥ 30%	PD-L1^+^ TC, 16% mPD-L1^+^ MEC, 27%	PD-L1^+^ tumor cells correlated with poor survivals.
Fang X. [51]	76	(SP142, ZSGB-BIO)	PD-L1^+^ ≥ 10%	PD-L1^+^ TC, 26.3% mPD-L1^+^ MEC, 27%	PD-L1^+^ tumor cells correlated with worse outcome in univariate analysis, but not multivariate analysis.
Hu L.Y. [56]	204	(Cell Signal tech)	PD-L1^+^ ≥ 10%	PD-L1^+^ TC, 49.0% mPD-L1^+^ MEC, 21.6%	PD-L1^+^ in tumor cells was an independent risk factor with poor OS.
Dong L. [57]	100	Polyclonal Ab (Abcam ab153991)	PD-L1^+^ ≥ 5%	PD-L1^+^ TC, 54.0%	PD-L1^+^ tumor cells were associated with poor outcomes.
Kiyasu J. [43]	1253	Monoclonal Ab (EPR1161)	PD-L1^+^ ≥ 30% mPD-L1^+^ ≥ 20%	PD-L1^+^ TC, 10.5% mPD-L1^+^ MEC, 15.3%	PD-L1^+^ was significantly associated with B symptoms, high IPI risk group and non-GC type. PD-L1^+^ PAX5^+^ tumor cells correlated with inferior OS.
**Data associated with sPD-L1 level**					
Rossille D. [58]	288	PDCD1LG1 ELISA	Cut-off value, 1.52 ng/mL	Elevated, 50.7%	Elevated sPD-L1 was associated with poor prognosis.
Rossille D. [59]	225	PDCD1LG1 ELISA	Cut-off, median value	Elevated, 50%	High sPD-L1 was an adverse prognostic factor.
Fest T. [60]	288	PDCD1LG1 ELISA	Cut-off, 95th percentile	ND	High sPD-L1 was associated with inferior OS in DLBCL patients treated immuno-chemotherapy.
Keane C. [61]	158	PDCD1LG1 ELISA	ND	ND	sPD-L1 was not significantly associated with prognosis.

sPD-L1, soluble PD-L1; Ab, antibody; TIL, tumor infiltrating lymphocyte; EN, extranodal; TC, tumor cell; MEC, microenvironment cell; PFS, progression-free survival; OS, overall survival; ND, not described; EFS, event-free survival; IPI, international prognostic index; GC, germinal center; mPD-L1; microenvironmental PD-L1.

**Table 2:** Clinical data of PD-1 blockade in diffuse large B cell lymphoma

Phase (Reference)	Study Design and Dose	Patients (Number)	Response (%)	Side Effects (%)	Survivals (Months)
**Nivolumab**					
Phase 1b [74]	1–3 mg/kg every 2 weeks	DLBCL, 11	ORR, 36/CR, 18	All AE—71 G3–4 AE—26	PFS, 1.8
Phase 2 [75]	3 mg/kg/2 weeks; ASCT-failed, median 4 doses; ASCT-ineligible, median 3 doses	DLBCL, 121; ASCT-failed, 87; ASCT-ineligible, 34	ASCT failed (median 9 months); ORR, 10 ASCT ineligible (median 6 months); ORR, 3	G3–4 AE—24	ASCT-failed—PFS, 1.9; OS, 12.2 ASCT-ineligible—PFS, 1.4; OS, 5.8
**Pembrolizumab**					
Phase 2 [76]	200 mg every 3 weeks	RT, 9	ORR, 44/CR, 11	All AE—100 G3–4 AE—60	PFS, 5.4/OS, 10.7

RT, Richter’s transformation; ORR, overall response rate; CR, complete response; AE, adverse effect; G3–4, grade 3–4; PFS, progression-free survival; OS, overall survival; DLBCL, diffuse large B cell lymphoma; ASCT, autologous stem cell transplantation.

## References

[1] Menon M.P., Pittaluga S., Jaffe E.S. (2012). The histological and biological spectrum of diffuse large B-cell lymphoma in the World Health Organization classification. Cancer J..

[2] Campo E., Swerdlow S.H., Harris N.L., Pileri S., Stein H., Jaffe E.S. (2011). The 2008 WHO classification of lymphoid neoplasms and beyond: Evolving concepts and practical applications. Blood.

[3] Feugier P., Van Hoof A., Sebban C., Solal-Celigny P., Bouabdallah R., Fermé C., Christian B., Lepage E., Tilly H., Morschhauser F. (2005). Long-term results of the R-CHOP study in the treatment of elderly patients with diffuse large B-cell lymphoma: A study by the Groupe d’Etude des Lymphomes de l’Adulte. J. Clin. Oncol..

[4] Greenwald R.J., Freeman G.J., Sharpe A.H. (2005). The B7 family revisited. Annu. Rev. Immunol..

[5] Fife B.T., Bluestone J.A. (2008). Control of peripheral T-cell tolerance and autoimmunity via the CTLA-4 and PD-1 pathways. Immunol. Rev..

[6] Keir M.E., Butte M.J., Freeman G.J., Sharpe A.H. (2008). PD-1 and its ligands in tolerance and immunity. Annu. Rev. Immunol..

[7] Zhang X., Schwartz J.C., Guo X., Bhatia S., Cao E., Lorenz M., Cammer M., Chen L., Zhang Z.Y., Edidin M.A. (2004). Structural and functional analysis of the costimulatory receptor programmed death-1. Immunity.

[8] Bennett F., Luxenberg D., Ling V., Wang I.M., Marquette K., Lowe D., Khan N., Veldman G., Jacobs K.A., Valge-Archer V.E. (2003). Program death-1 engagement upon TCR activation has distinct effects on costimulation and cytokine-driven proliferation: Attenuation of ICOS, IL-4, and IL-21, but not CD28, IL-7, and IL-15 responses. J. Immunol..

[9] Daëron M., Jaeger S., Du Pasquier L., Vivier E. (2008). Immunoreceptor tyrosine-based inhibition motifs: A quest in the past and future. Immunol. Rev..

[10] El Firar A., Voisin T., Rouyer-Fessard C., Ostuni M.A., Couvineau A., Laburthe M. (2009). Discovery of a functional immunoreceptor tyrosine-based switch motif in a 7-transmembrane-spanning receptor: Role in the orexin receptor OX1R-driven apoptosis. FASEB J..

[11] Dong H., Zhu G., Tamada K., Chen L. (1999). B7-H1, a third member of the B7 family, co-stimulates T-cell proliferation and interleukin-10 secretion. Nat. Med..

[12] Youngnak P., Kozono Y., Kozono H., Iwai H., Otsuki N., Jin H., Omura K., Yagita H., Pardoll D.M., Chen L. (2003). Differential binding properties of B7-H1 and B7-DC to programmed death-1. Biochem. Biophys. Res. Commun..

[13] Andorsky D.J., Yamada R.E., Said J., Pinkus G.S., Betting D.J., Timmerman J.M. (2011). Programmed death ligand 1 is expressed by non-Hodgkin lymphomas and inhibits the activity of tumor-associated T cells. Clin. Cancer Res..

[14] Laurent C., Charmpi K., Gravelle P., Tosolini M., Franchet C., Ysebaert L., Brousset P., Bidaut A., Ycart B., Fournié J.J. (2015). Several immune escape patterns in non-Hodgkin’s lymphomas. Oncoimmunology.

[15] Chen L., Flies D.B. (2013). Molecular mechanisms of T cell co-stimulation and co-inhibition. Nat. Rev. Immunol..

[16] Chemnitz J.M., Parry R.V., Nichols K.E., June C.H., Riley J.L. (2004). SHP-1 and SHP-2 associate with immunoreceptor tyrosine-based switch motif of programmed death 1 upon primary human T cell stimulation, but only receptor ligation prevents T cell activation. J. Immunol..

[17] Sheppard K.A., Fitz L.J., Lee J.M., Benander C., George J.A., Wooters J., Qiu Y., Jussif J.M., Carter L.L., Wood C.R. (2004). PD-1 inhibits T-cell receptor induced phosphorylation of the ZAP70/CD3zeta signalosome and downstream signaling to PKCtheta. FEBS Lett..

[18] Parry R.V., Chemnitz J.M., Frauwirth K.A., Lanfranco A.R., Braunstein I., Kobayashi S.V., Linsley P.S., Thompson C.B., Riley J.L. (2005). CTLA-4 and PD-1 receptors inhibit T-cell activation by distinct mechanisms. Mol. Cell. Biol..

[19] Georgiou K., Chen L., Berglund M., Ren W., de Miranda N.F., Lisboa S., Fangazio M., Zhu S., Hou Y., Wu K. (2016). Genetic basis of PD-L1 overexpression in diffuse large B-cell lymphomas. Blood.

[20] Green M.R., Monti S., Rodig S.J., Juszczynski P., Currie T., O’Donnell E., Chapuy B., Takeyama K., Neuberg D., Golub T.R. (2010). Integrative analysis reveals selective 9p24.1 amplification, increased PD-1 ligand expression, and further induction via JAK2 in nodular sclerosing Hodgkin lymphoma and primary mediastinal large B-cell lymphoma. Blood.

[21] Chen B.J., Chapuy B., Ouyang J., Sun H.H., Roemer M.G., Xu M.L., Yu H., Fletcher C.D., Freeman G.J., Shipp M.A. (2013). PD-L1 expression is characteristic of a subset of aggressive B-cell lymphomas and virus-associated malignancies. Clin. Cancer Res..

[22] Gupta M., Han J.J., Stenson M., Maurer M., Wellik L., Hu G., Ziesmer S., Dogan A., Witzig T.E. (2012). Elevated serum IL-10 levels in diffuse large B-cell lymphoma: A mechanism of aberrant JAK2 activation. Blood.

[23] Blank C., Brown I., Pefdterson A.C., Spiotto M., Iwai Y., Honjo T., Gajewski T.F. (2004). PD-L1/B7H-1 inhibits the effector phase of tumor rejection by T cell receptor (TCR) transgenic CD8+ T cells. Cancer Res..

[24] Spranger S., Spaapen R.M., Zha Y., Williams J., Meng Y., Ha T.T., Gajewski T.F. (2013). Up-regulation of PD-L1, IDO, and T(regs) in the melanoma tumor microenvironment is driven by CD8 (+) T cells. Sci. Transl. Med..

[25] Croker B.A., Kiu H., Nicholson S.E. (2008). SOCS Regulation of the JAK/STAT Signaling Pathway. Semin. Cell Dev. Biol..

[26] Yoshikawa H., Matsubara K., Qian G.S., Jackson P., Groopman J.D., Manning J.E., Harris C.C., Herman J.G. (2001). SOCS-1, a negative regulator of the JAK/STAT pathway, is silenced by methylation in human hepatocellular carcinoma and shows growth-suppression activity. Nat. Genet..

[27] Weniger M.A., Melzner I., Menz C.K., Wegener S., Bucur A.J., Dorsch K., Mattfeldt T., Barth T.F., Möller P. (2006). Mutations of the tumor suppressor gene SOCS-1 in classical Hodgkin lymphoma are frequent and associated with nuclear phospho-STAT5 accumulation. Oncogene.

[28] Melzner I., Bucur A.J., Brüderlein S., Dorsch K., Hasel C., Barth T.F., Leithäuser F., Möller P. (2005). Biallelic mutation of SOCS-1 impairs JAK2 degradation and sustains phospho-JAK2 action in the MedB-1 mediastinal lymphoma line. Blood.

[29] Chen L., Gibbons D.L., Goswami S., Cortez M.A., Ahn Y.H., Byers L.A., Zhang X., Yi X., Dwyer D., Lin W. (2014). Metastasis is regulated via microRNA-200/ZEB1 axis control of tumour cell PD-L1 expression and intratumoral immunosuppression. Nat. Commun..

[30] Holla S., Stephenvictor E., Prakhar P., Sharma M., Saha C., Udupa V., Kaveri S.V., Bayry J. (2016). Mycobacteria-responsive sonic hedgehog signaling mediates programmed death-ligand 1- and prostaglandin E2-induced regulatory T cell expansion. Sci. Rep..

[31] Xu S., Tao Z., Hai B., Liang H., Shi Y., Wang T., Song W., Chen Y., Ouyang J., Chen J. (2016). MiR-424(322) reverses chemoresistance via T-cell immune response activation by blocking the PD-L1 immune checkpoint. Nat. Commun..

[32] Lee S.J., Jang B.C., Lee S.W., Yang Y.I., Suh S.I., Park Y.M., Oh S., Shin J.G., Yao S., Chen L. (2006). Interferon regulatory factor-1 is prerequisite to the constitutive expression and IFNgamma-induced upregulation of B7-H1 (CD274). FEBS Lett..

[33] Dorand R.D., Nthale J., Myers J.T., Barkauskas D.S., Avril S., Chirieleison S.M., Pareek T.K., Abbott D.W., Stearns D.S., Letterio J.J. (2016). Cdk5 disruption attenuates tumor PD-L1 expression and promotes antitumor immunity. Science.

[34] Lim S.O., Li C.W., Xia W., Cha J.H., Chan L.C., Wu Y., Chang S.S., Lin W.C., Hsu J.M., Hsu Y.H. (2016). Deubiquitination and Stabilization of PD-L1 by CSN5. Cancer Cell.

[35] He B., Yan F., Wu C. (2018). Overexpressed miR-195 attenuated immune escape of diffuse large B-cell lymphoma by targeting PD-L1. Biomed. Pharmacother..

[36] Armand P. (2015). Immune checkpoint blockade in hematologic malignancies. Blood.

[37] Green M.R., Rodig S., Juszczynski P., Ouyang J., Sinha P., O’Donnell E., Neuberg D., Shipp M.A. (2012). Constitutive AP-1 activity and EBV infection induce PD-L1 in Hodgkin lymphomas and posttransplant lymphoproliferative disorders: Implications for targeted therapy. Clin. Cancer Res..

[38] Mathas S., Hinz M., Anagnostopoulos I., Krappmann D., Lietz A., Jundt F., Bommert K., Mechta-Grigoriou F., Stein H., Dörken B. (2002). Aberrantly expressed c-Jun and JunB are a hallmark of Hodgkin lymphoma cells, stimulate proliferation and synergize with NF-kappa B. EMBO J..

[39] Rawlings D.J., Schwartz M.A., Jackson S.W., Meyer-Bahlburg A. (2012). Integration of B cell responses through Toll-like receptors and antigen receptors. Nat. Rev. Immunol..

[40] Akira S., Takeda K. (2004). Toll-like receptor signaling. Nat. Rev. Immunol..

[41] Lin S.C., Lo Y.C., Wu H. (2010). Helical assembly in the MyD88-IRAK4-IRAK2 complex in TLR/IL-1R signaling. Nature.

[42] Ngo V.N., Young R.M., Schmitz R., Jhavar S., Xiao W., Lim K.H., Kohlhammer H., Xu W., Yang Y., Zhao H. (2011). Oncogenically active MYD88 mutations in human lymphoma. Nature.

[43] Kiyasu J., Miyoshi H., Hirata A., Arakawa F., Ichikawa A., Niino D., Sugita Y., Yufu Y., Choi I., Abe Y. (2015). Expression of programmed cell death ligand 1 is associated with poor overall survival in patients with diffuse large B-cell lymphoma. Blood.

[44] Twa D.D., Chan F.C., Ben-Neriah S., Woolcock B.W., Mottok A., Tan K.L., Slack G.W., Gunawardana J., Lim R.S., McPherson A.W. (2014). Genomic rearrangements involving programmed death ligands are recurrent in primary mediastinal large B-cell lymphoma. Blood.

[45] Swerdlow S.H., Campo E., Pileri S.A., Harris N.L., Stein H., Siebert R., Advani R., Ghielmini M., Salles G.A., Zelenetz A.D. (2016). The 2016 revision of the World Health Organization classification of lymphoid neoplasms. Blood.

[46] Qiu H., Hu X., Gao L., Chen L., Chen J., Yuan J., Huang C., Xu X., Yang J. (2017). Interleukin 10 enhanced CD8+ T cell activity and reduced CD8+ T cell apoptosis in patients with diffuse large B cell lymphoma. Exp. Cell Res..

[47] Choi J.W., Kim Y., Lee J.H., Kim Y.S. (2013). MYD88 expression and L265P mutation in diffuse large B-cell lymphoma. Hum. Pathol..

[48] Dubois S., Viailly P.J., Bohers E., Bertrand P., Ruminy P., Marchand V., Maingonnat C., Mareschal S., Picquenot J.M., Penther D. (2017). Biological and Clinical Relevance of Associated Genomic Alterations in MYD88 L265P and non-L265P-Mutated Diffuse Large B-Cell Lymphoma: Analysis of 361 Cases. Clin. Cancer Res..

[49] Ahearne M.J., Bhuller K., Hew R., Ibrahim H., Naresh K., Wagner S.D. (2014). Expression of PD-1 (CD279) and FoxP3 in diffuse large B-cell lymphoma. Virchows Arch..

[50] Kwon D., Kim S., Kim P.J., Go H., Nam S.J., Paik J.H., Kim Y.A., Kim T.M., Heo D.S., Kim C.W. (2016). Clinicopathological analysis of programmed cell death 1 and programmed cell death ligand 1 expression in the tumour microenvironments of diffuse large B cell lymphomas. Histopathology.

[51] Fang X., Xiu B., Yang Z., Qiu W., Zhang L., Zhang S., Wu Y., Zhu X., Chen X., Xie S. (2017). The expression and clinical relevance of PD-1, PD-L1, and TP63 in patients with diffuse large B-cell lymphoma. Medicine (Baltimore).

[52] Muenst S., Hoeller S., Willi N., Dirnhofera S., Tzankov A. (2010). Diagnostic and prognostic utility of PD-1 in B cell lymphomas. Dis. Mark..

[53] Cohen M., Vistarop A.G., Huaman F., Narbaitz M., Metrebian F., De Matteo E., Preciado M.V., Chabay P.A. (2017). Cytotoxic response against Epstein Barr virus coexists with diffuse large B-cell lymphoma tolerogenic microenvironment: Clinical features and survival impact. Sci. Rep..

[54] Siddiqi I.N., Thodima V., Friedman J., Violeta A., Tulpule A., Shaknovich R., Houldsworth J. (2016). PD-L1 Expression Identifies High Risk Diffuse Large B-Cell Lymphoma and Is Associated with Several Genomic Markers. Blood.

[55] Xing W., Dresser K., Zhang R., Evens A.M., Yu H., Woda B.A., Chen B.J. (2016). PD-L1 expression in EBV-negative diffuse large B-cell lymphoma: Clinicopathologic features and prognostic implications. Oncotarget.

[56] Hu L.Y., Xu X.L., Rao H.L., Chen J., Lai R.C., Huang H.Q., Jiang W.Q., Lin T.Y., Xia Z.J., Cai Q.Q. (2017). Expression and clinical value of programmed cell death-ligand 1 (PD-L1) in diffuse large B cell lymphoma: A retrospective study. Chin. J. Cancer.

[57] Dong L., Lv H., Li W., Song Z., Li L., Zhou S., Qiu L., Qian Z., Liu X., Feng L. (2016). Co-expression of PD-L1 and p-AKT is associated with poor prognosis in diffuse large B-cell lymphoma via PD-1/PD-L1 axis activating intracellular AKT/mTOR pathway in tumor cells. Oncotarget.

[58] Rossille D., Gressier M., Damotte D., Maucort-Boulch D., Pangault C., Semana G., Le Gouill S., Haioun C., Tarte K., Lamy T. (2014). High level of soluble programmed cell death ligand 1 in blood impacts overall survival in aggressive diffuse large B-Cell lymphoma: Results from a French multicenter clinical trial. Leukemia.

[59] Rossille D., Azzaoui I., Feldman A.L., Maurer M.J., Labouré G., Parrens M., Pangault C., Habermann T.M., Ansell S.M., Link B.K. (2017). Soluble programmed death-ligand 1 as a prognostic biomarker for overall survival in patients with diffuse large B-cell lymphoma: A replication study and combined analysis of 508 patients. Leukemia.

[60] Fest T., Rossille D., Gressier M., Maucort-Boulch D., Damotte D., Pangault C., Le Gouill S., Tarte K., Lamy T., Milpied N. (2013). Blood Soluble PD-L1 Protein In Aggressive Diffuse Large B-Cell Lymphoma Impacts patient’s Overall Survival. Blood.

[61] Keane C., Vari F., Hertzberg M., Cao K.A., Green M.R., Han E., Seymour J.F., Hicks R.J., Gill D., Crooks P. (2015). Ratios of T-cell immune effectors and checkpoint molecules as prognostic biomarkers in diffuse large B-cell lymphoma: A population-based study. Lancet Haematol..

[62] Thompson R.H., Kuntz S.M., Leibovich B.C., Dong H., Lohse C.M., Webster W.S., Sengupta S., Frank I., Parker A.S., Zincke H. (2006). Tumor B7-H1 is associated with poor prognosis in renal cell carcinoma patients with long-term follow-up. Cancer Res..

[63] Kim J.R., Moon Y.J., Kwon K.S., Bae J.S., Wagle S., Kim K.M., Park H.S., Lee H., Moon W.S., Chung M.J. (2013). Tumor infiltrating PD1-positive lymphocytes and the expression of PD-L1 predict poor prognosis of soft tissue sarcomas. PLoS ONE.

[64] Carreras J., Lopez-Guillermo A., Roncador G., Villamor N., Colomo L., Martinez A., Hamoudi R., Howat W.J., Montserrat E., Campo E. (2009). High numbers of tumor-infiltrating programmed cell death 1-positive regulatory lymphocytes are associated with improved overall survival in follicular lymphoma. J. Clin. Oncol..

[65] Wahlin B.E., Aggarwal M., Montes-Moreno S., Gonzalez L.F., Roncador G., Sanchez-Verde L., Christensson B., Sander B., Kimby E. (2010). A unifying microenvironment model in follicular lymphoma: Outcome is predicted by programmed death-1-positive, regulatory, cytotoxic, and helper T cells and macrophages. Clin. Cancer Res..

[66] Xu-Monette Z.Y., Zhou J., Young K.H. (2018). PD-1 expression and clinical PD-1 blockade in B-cell lymphomas. Blood.

[67] Pedoeem A., Azoulay-Alfaguter I., Strazza M., Silverman G.J., Mor A. (2014). Programmed death-1 pathway in cancer and autoimmunity. Clin. Immunol..

[68] Xu D., Hu J., Xu S., De Bruyne E., Menu E., Van Camp B., Vanderkerken K., Van Valckenborgh E. (2012). Dll1/Notch activation accelerates multiple myeloma disease development by promoting CD138+ MM-cell proliferation. Leukemia.

[69] Zhang J., Medeiros L.J., Young K.H. (2018). Cancer Immunotherapy in Diffuse Large B-Cell Lymphoma. Front Oncol..

[70] Bryan L.J., Gordon L. (2015). Blocking tumor escape in hematologic malignancies: The anti-PD-1 strategy. Blood Rev..

[71] Hodi F.S., Chiarion-Sileni V., Gonzalez R., Grob J.J., Rutkowski P., Cowey C.L., Lao C.D., Schadendorf D., Wagstaff J., Dummer R. (2018). Nivolumab plus ipilimumab or nivolumab alone versus ipilimumab alone in advanced melanoma (CheckMate 067): 4-year outcomes of a multicentre, randomised, phase 3 trial. Lancet Oncol..

[72] Antonia S.J., López-Martin J.A., Bendell J., Ott P.A., Taylor M., Eder J.P., Jäger D., Pietanza M.C., Le D.T., de Braud F. (2016). Nivolumab alone and nivolumab plus ipilimumab in recurrent small-cell lung cancer (CheckMate 032): A multicentre, open-label, phase 1/2 trial. Lancet Oncol..

[73] Daver N., Garcia-Manero G., Basu S., Boddu P.C., Alfayez M., Cortes J.E., Konopleva M., Ravandi-Kashani F., Jabbour E., Kadia T. (2018). Efficacy, Safety, and Biomarkers of Response to Azacitidine and Nivolumab in Relapsed/Refractory Acute Myeloid Leukemia: A Nonrandomized, Open-Label, Phase II Study. Cancer Discov..

[74] Lesokhin A.M., Ansell S.M., Armand P., Scott E.C., Halwani A., Gutierrez M., Millenson M.M., Cohen A.D., Schuster S.J., Lebovic D. (2016). Nivolumab in Patients With Relapsed or Refractory Hematologic Malignancy: Preliminary Results of a Phase Ib Study. J. Clin. Oncol..

[75] Ansell S.M., Minnema M.C., Johnson P., Timmerman J.M., Armand P., Shipp M.A., Rodig S.J., Ligon A.H., Roemer M.G.M., Reddy N. (2019). Nivolumab for Relapsed/Refractory Diffuse Large B-Cell Lymphoma in Patients Ineligible for or Having Failed Autologous Transplantation: A Single-Arm, Phase II Study. J. Clin. Oncol..

[76] Ding W., LaPlant B.R., Call T.G., Parikh S.A., Leis J.F., He R., Shanafelt T.D., Sinha S., Le-Rademacher J., Feldman A.L. (2017). Pembrolizumab in patients with CLL and Richter transformation or with relapsed CLL. Blood.

[77] Merck Sharp & Dohme Corp (2018). KEYTRUDA® (Pembrolizumab) for Injection, for Intravenous Use, 08.

[78] Merck Sharp & Dohme Limited (2017). Keytruda 50 mg Powder for Concentrate for Solution for Infusion.

[79] Jeanson A., Barlesi F. (2017). MEDI 4736 (durvalumab) in non-small cell lung cancer. Expert Opin. Biol. Ther..

[80] Bellmunt J., Powles T., Vogelzang N.J. (2017). A review on the evolution of PD-1/PD-L1 immunotherapy for bladder cancer: The future is now. Cancer Treat. Rev..

[81] Shah N.J., Kelly W.J., Liu S.V., Choquette K., Spira A. (2018). Product review on the Anti-PD-L1 antibody atezolizumab. Hum. Vaccines Immunother..

[82] Rao A., Patel M.R. (2019). A review of avelumab in locally advanced and metastatic bladder cancer. Ther. Adv. Urol..

[83] Champiat S., Dercle L., Ammari S., Massard C., Hollebecque A., Postel-Vinay S., Chaput N., Eggermont A., Marabelle A., Soria J.C. (2017). Hyperprogressive Disease Is a New Pattern of Progression in Cancer Patients Treated by Anti-PD-1/PD-L1. Clin. Cancer Res..

[84] Kato S., Goodman A., Walavalkar V., Barkauskas D.A., Sharabi A., Kurzrock R. (2017). Hyperprogressors after Immunotherapy: Analysis of Genomic Alterations Associated with Accelerated Growth Rate. Clin. Cancer Res..

[85] Nguyen L.T., Ohashi P.S. (2015). Clinical blockade of PD1 and LAG3—Potential mechanisms of action. Nat. Rev. Immunol..

[86] Ghiotto M., Gauthier L., Serriari N., Pastor S., Truneh A., Nunès J.A., Olive D. (2010). PD-L1 and PD-L2 differ in their molecular mechanisms of interaction with PD-1. Int. Immunol..

[87] Le D.T., Uram J.N., Wang H., Bartlett B.R., Kemberling H., Eyring A.D., Skora A.D., Luber B.S., Azad N.S., Laheru D. (2015). PD-1 Blockade in tumors with mismatch-repair deficiency. N. Engl. J. Med..

[88] Dudley J.C., Lin M.T., Le D.T., Eshleman J.R. (2016). Microsatellite instability as a biomarker for PD-1 blockade. Clin. Cancer Res..

